# Reduction in Renal Heme Oxygenase-1 Is Associated with an Aggravation of Kidney Injury in Shiga Toxin-Induced Murine Hemolytic-Uremic Syndrome

**DOI:** 10.3390/toxins16120543

**Published:** 2024-12-14

**Authors:** Antonio N. Mestekemper, Wiebke Pirschel, Nadine Krieg, Maria K. Paulmann, Christoph Daniel, Kerstin Amann, Sina M. Coldewey

**Affiliations:** 1 Department of Anesthesiology and Intensive Care Medicine, Jena University Hospital, Am Klinikum 1, 07747 Jena, Germany; antonio.mestekemper@charite.de (A.N.M.); nadine.krieg@med.uni-jena.de (N.K.); maria.paulmann@med.uni-jena.de (M.K.P.); 2 Septomics Research Center, Jena University Hospital, 07745 Jena, Germany; 3 Department of Nephropathology, Friedrich-Alexander University (FAU) Erlangen-Nürnberg, 91054 Erlangen, Germany; christoph.daniel@uk-erlangen.de (C.D.); kerstin.amann@uk-erlangen.de (K.A.); 4 Center for Sepsis Control and Care (CSCC), Jena University Hospital, 07747 Jena, Germany

**Keywords:** hemolytic-uremic syndrome, Shiga toxin, heme oxygenase-1, mouse model, kidney injury

## Abstract

Hemolytic-uremic syndrome (HUS) is a systemic complication of an infection with Shiga toxin (Stx)-producing enterohemorrhagic *Escherichia coli*, primarily leading to acute kidney injury (AKI) and microangiopathic hemolytic anemia. Although free heme has been found to aggravate renal damage in hemolytic diseases, the relevance of the heme-degrading enzyme heme oxygenase-1 (HO-1, encoded by *Hmox1*) in HUS has not yet been investigated. We hypothesized that HO-1*,* also important in acute phase responses in damage and inflammation, contributes to renal pathogenesis in HUS. The effect of tamoxifen-induced *Hmox1* gene deletion on renal HO-1 expression, disease progression and AKI was investigated in mice 7 days after HUS induction. Renal HO-1 levels were increased in Stx-challenged mice with tamoxifen-induced *Hmox1* gene deletion (Hmox1^R26Δ/Δ^) and control mice (Hmox1^lox/lox^). This HO-1 induction was significantly lower (−43%) in Hmox1^R26Δ/Δ^ mice compared to Hmox1^lox/lox^ mice with HUS. Notably, the reduced renal HO-1 expression was associated with an exacerbation of kidney injury in mice with HUS as indicated by a 1.7-fold increase (*p* = 0.02) in plasma neutrophil gelatinase-associated lipocalin (NGAL) and a 1.3-fold increase (*p* = 0.06) in plasma urea, while other surrogate parameters for AKI (e.g., periodic acid Schiff staining, kidney injury molecule-1, fibrin deposition) and general disease progression (HUS score, weight loss) remained unchanged. These results indicate a potentially protective role of HO-1 in the pathogenesis of Stx-mediated AKI in HUS.

## 1. Introduction

Hemolytic-uremic syndrome (HUS) is a rare but life-threatening complication of an infection with Shiga toxin (Stx)-producing enterohemorrhagic *Escherichia coli* (STEC). The bacterial toxin Stx is a main contributor to the pathogenesis of STEC-HUS [1,2]. There are different types of Stx (Stx1 and Stx2), of which particularly Stx2 is associated with the development of HUS [1,3]. Stx can enter cells via various mechanisms. Primarily, Stx binds to globotriaosylceramide (Gb3), which is highly present on renal endothelial cells, or enters these cells independent of Gb3 via microvesicles [4]. Intracellular Stx leads to endothelial apoptosis and renal tissue damage by interfering with protein synthesis [4]. Renal histopathology of STEC-HUS patients typically reveals thrombotic microangiopathy (TMA) which is indicated by swollen and detached endothelial cells, intravascular clot formation and fibrin depositions [5]. Clinically, HUS manifests as thrombotic-microangiopathic hemolytic anemia and acute kidney injury (AKI). To date, many pathophysiological aspects are still not fully understood and current therapy is mainly supportive [6]. Since severe cases of HUS frequently require dialysis and pose the risk of progression to chronic and end-stage kidney disease [4,7], it is important to further elucidate the underlying mechanisms involved in HUS pathogenesis and to identify molecular targets for new potential therapeutic approaches.

There is evidence that free heme propagates disease progression in hemolytic disorders and life-threatening infections [8,9,10], including sepsis [8], malaria [10] and sickle cell disease [11,12,13,14]. The degradation of heme into equimolar amounts of ferrous iron, carbon monoxide (CO) and biliverdin is catalyzed by heme oxygenases (HO) which are intracellular enzymes found in humans and mice [15]. While the ubiquitously expressed HO-1 isoform is induced during inflammatory or oxidative conditions, HO-2 is constitutively expressed in various tissues [16]. Nevertheless, it has been demonstrated that HO-2 is less effective in exerting a cytoprotective function against substantial quantities of free heme during hemolysis [17,18], whereas HO-1 allows the efficient recycling of heme-bound iron under hemolytic conditions which would otherwise cause oxidative stress [19] and inflammation [8,20]. The induction of HO-1, encoded by the gene *heme oxygenase-1* (*Hmox1)*, is associated with various anti-inflammatory, anti-proliferative as well as anti-apoptotic responses in answer to diverse stress conditions [16,17]. In the past, enormous efforts have been made to investigate these cytoprotective effects of HO-1, considering it as a therapeutic target in distinct diseases [16] such as malaria [21,22] and sepsis [8]. Hitherto, the role of HO-1 in HUS pathology has been poorly studied. We have recently demonstrated that the extracellular hemoglobin scavenger haptoglobin exerts protective functions, and observed that HO-1 is induced in kidneys of mice subjected to Stx [23]. Furthermore, Wijnsma et al. reported that free heme and HO-1 are increased in the plasma of STEC-HUS patients with severe hemolysis [24]. To test the hypothesis that induction of HO-1 has an impact on the pathogenesis and progression of HUS, we investigated the effects of *Hmox1* deficiency on clinical outcome, kidney injury and TMA in a mouse model of HUS-like disease.

## 2. Results

### 2.1. Hmox1 Expression in the Blood Prior to HUS Induction and Renal HO-1 Levels in Hmox1^lox/lox^ and Hmox1^R26Δ/Δ^ Mice with HUS

**Figure 1:**
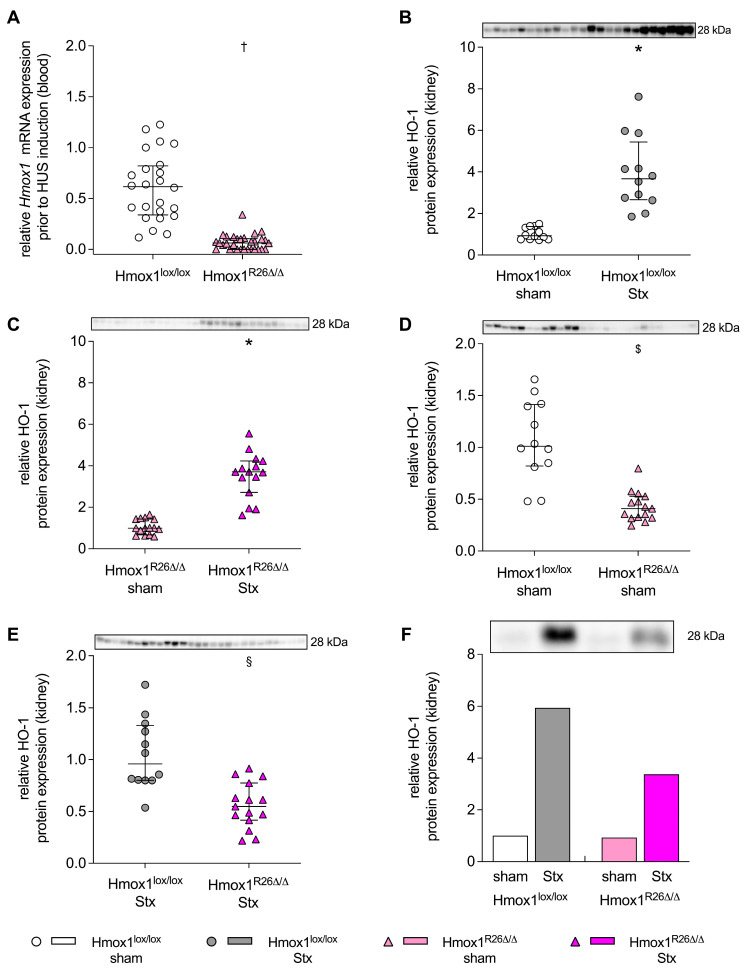
*Hmox1* gene expression in the blood of Hmox1^lox/lox^ and Hmox1^R26Δ/Δ^ mice and heme oxygenase-1 (HO-1) protein expression in the kidneys of Hmox1^lox/lox^ and Hmox1^R26Δ/Δ^ mice with experimental hemolytic-uremic syndrome (HUS). (**A**) Relative *Hmox1* expression in the blood of Hmox1^lox/lox^ and Hmox1^R26Δ/Δ^ mice prior to HUS induction (Hmox1^lox/lox^: *n* = 24 per group; Hmox1^R26Δ/Δ^: *n* = 30 per group). (**B**–**E**) HO-1 expression in the kidneys of Hmox1^lox/lox^ and Hmox1^R26Δ/Δ^ sham mice as well as Shiga toxin (Stx)-challenged Hmox1^lox/lox^ and Hmox1^R26Δ/Δ^ mice at day 7 after initial HUS induction (Hmox1^lox/lox^: *n* = 12 per group, Hmox1^R26Δ/Δ^: *n* = 15 per group): (**B**) renal HO-1 expression in Hmox1^lox/lox^ sham and Stx-challenged Hmox1^lox/lox^ mice; (**C**) renal HO-1 expression in Hmox1^R26Δ/Δ^ sham and Stx-challenged Hmox1^R26Δ/Δ^ mice; (**D**) renal HO-1 expression in Hmox1^lox/lox^ and Hmox1^R26Δ/Δ^ sham mice; (**E**) renal HO-1 expression in Stx-challenged Hmox1^lox/lox^ and Hmox1^R26Δ/Δ^ mice. (**B**–**E**) Data are presented relative to the mean of (**B**,**D**) Hmox1^lox/lox^ sham mice, (**C**) Hmox1^R26Δ/Δ^ sham mice and (**E**) Hmox1^lox/lox^ Stx mice and are expressed as (**A**–**E**) scatter dot plots with median (interquartile range) for *n* observations. ^†^ *p* < 0.05 Hmox1^lox/lox^ vs. Hmox1^R26Δ/Δ^, * *p* < 0.05 vs. corresponding sham group, ^$^ *p* < 0.05 vs. Hmox1^lox/lox^ sham group, ^§^ *p* < 0.05 vs. Hmox1^lox/lox^ Stx group (Mann–Whitney *U* test). (**F**) For illustration, renal HO-1 protein expressions in pooled samples from all mice in all groups studied are shown relative to each other (pooled samples from Hmox1^lox/lox^ sham mice (*n* = 12), Hmox1^R26∆/∆^ sham mice (*n* = 15), Stx-challenged Hmox1^lox/lox^ mice (*n* = 12), Stx-challenged Hmox1^R26Δ/Δ^ mice, (*n* = 15)). Data are presented as bar graph relative to the pooled Hmox1^lox/lox^ sham group. No quantitative and thus statistical conclusions can be derived from this immunoblot, since the samples were pooled. All immunoblots are shown in larger format in the supplement (Supplementary Figure S1A–D).

After completion of the tamoxifen (TMX) diet and prior to the induction of HUS, *Hmox1* expression in the blood of Hmox1^R26∆/∆^ was decreased to 10% of that in Hmox1^lox/lox^ mice (Figure 1A; *p* < 0.0001). HO-1 levels in the kidneys of Stx-challenged Hmox1^lox/lox^ (Figure 1B; *n* = 12 per group) and Hmox1^R26∆/∆^ (Figure 1C; *n* = 15 per group) mice were significantly elevated 7 days after HUS induction compared to their corresponding sham group. However, renal HO-1 levels were 60% lower (*p* < 0.0001) in Hmox1^R26∆/∆^ sham mice (*n* = 15) compared to Hmox1^lox/lox^ sham mice (*n* = 12) (Figure 1D). Further, renal HO-1 expression was 43% lower (*p* = 0.0005) in Stx-challenged Hmox1^R26∆/∆^ mice (*n* = 15) compared to Stx-challenged Hmox1^lox/lox^ mice (*n* = 12) (Figure 1E). For illustrative purposes, Figure 1F shows the renal HO-1 levels of all groups relative to each other. However, no quantitative or statistical conclusions can be drawn from this immunoblot as the samples were pooled.

### 2.2. Effect of Hmox1 Deficiency on Clinical Presentation of Mice with HUS

**Figure 2:**
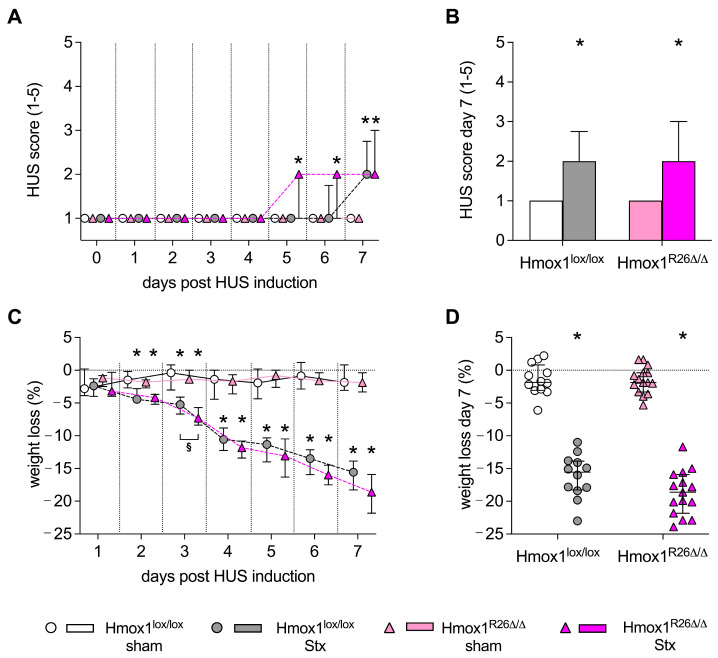
Clinical presentation of Hmox1^lox/lox^ and Hmox1^R26Δ/Δ^ mice with experimental hemolytic-uremic syndrome (HUS). (**A**–**D**) Experimental HUS was followed up for 7 days in Hmox1^lox/lox^ and Hmox1^R26Δ/Δ^ sham mice and Shiga toxin (Stx)-challenged Hmox1^lox/lox^ and Hmox1^R26Δ/Δ^ mice (Hmox1^lox/lox^: *n* = 12 per group, Hmox1^R26Δ/Δ^: *n* = 15 per group). (**A**) Disease progression indicated by HUS score (ranging from 1 = no signs of illness to 5 = dead) over 7 days and (**B**) significant changes in HUS score on day 7 in Hmox1^lox/lox^ and Hmox1^R26Δ/Δ^ sham mice and Stx-challenged Hmox1^lox/lox^ and Hmox1^R26Δ/Δ^ mice. (**C**) Progression of weight loss from day 1 to 7 and (**D**) significant changes in weight loss on day 7 in Hmox1^lox/lox^ and Hmox1^R26Δ/Δ^ sham mice compared to Stx-challenged Hmox1^lox/lox^ and Hmox1^R26Δ/Δ^ mice. Data are expressed as (**A**,**C**) dot plots, (**B**) bar graph and (**D**) scatter dot plot, with median and interquartile range for n observations. * *p* < 0.05 vs. corresponding sham group.

In order to evaluate the impact of renal HO-1 reduction on the development of HUS, we monitored the clinical presentation (HUS score, weight loss) and survival of Hmox1^R26∆/∆^ and Hmox1^lox/lox^ mice for a period of 7 days. All mice were observed to survive until the designated endpoint on day 7. The HUS score (see Supplementary Table S3) was used to continuously monitor disease progression, but was not used for diagnostic purposes. On day 5, Stx-challenged Hmox1^R26Δ/Δ^ mice showed clear signs of disease (indicated by a significant increase in the HUS score), while Stx-challenged Hmox1^lox/lox^ mice showed clear signs of disease on day 7 (Figure 2A). On day 7, the HUS score was significantly elevated in Stx-challenged Hmox1^lox/lox^ and Hmox1^R26Δ/Δ^ mice compared to their corresponding sham group (Figure 2B). Weight loss was observed to increase continuously in Stx-challenged Hmox1^lox/lox^ as well as Hmox1^R26Δ/Δ^ mice, becoming apparent on day 2 after the initial induction of HUS (Figure 2C). The weight loss on day 3 was slightly higher in Stx-challenged Hmox1^R26Δ/Δ^ mice (−7%) than in Stx-challenged Hmox1^lox/lox^ mice (−5%) (Figure 2C). On day 7, Hmox1^R26Δ/Δ^ and Hmox1^lox/lox^ mice with HUS demonstrated a significant reduction in weight compared to their corresponding sham group (18% and 15% respectively; *p* < 0.0001) (Figure 2D).

### 2.3. Effect of Hmox1 Deficiency on Kidney Injury in Mice with HUS

**Figure 3:**
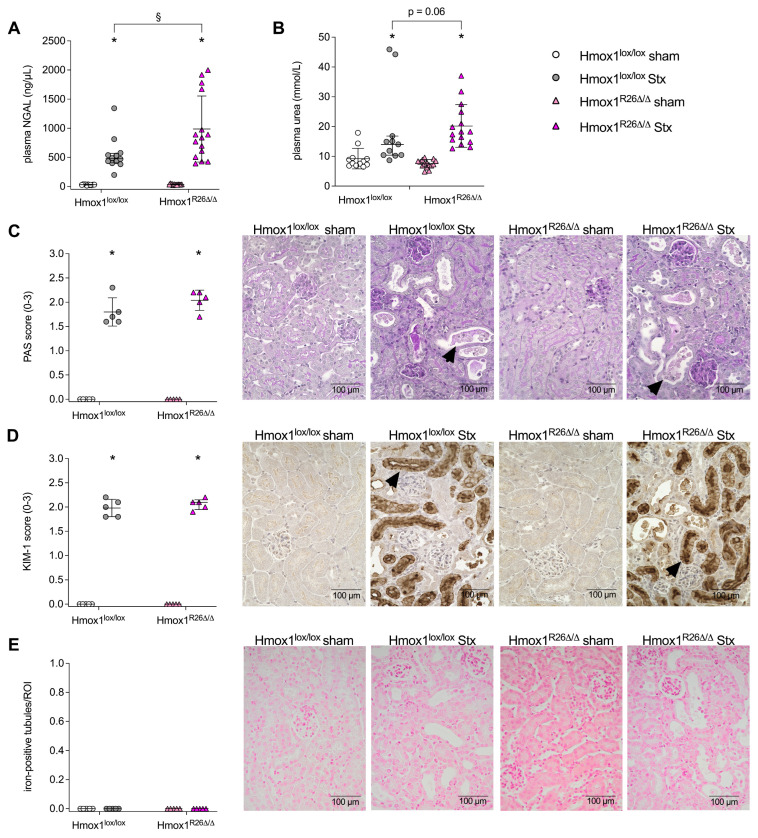
Kidney injury in Hmox1^lox/lox^ and Hmox1^R26Δ/Δ^ mice with experimental hemolytic-uremic syndrome (HUS). (**A**) Neutrophil gelatinase-associated lipocalin (NGAL) (Hmox1^lox/lox^ sham: *n* = 11, Hmox1^lox/lox^ Stx: *n* = 12, Hmox1^R26Δ/Δ^ sham: *n* = 14, Hmox1^R26Δ/Δ^ Stx: *n* = 15) and (**B**) urea plasma levels on day 7 in Hmox1^lox/lox^ and Hmox1^R26Δ/Δ^ sham mice and Stx-challenged Hmox1^lox/lox^ and Hmox1^R26Δ/Δ^ mice (Hmox1^lox/lox^ sham: *n* = 12, Hmox1^lox/lox^ Stx: *n* = 11, Hmox1^R26Δ/Δ^: *n* = 15 per group). Quantification and representative pictures of (**C**) periodic acid Schiff (PAS) reaction and immunohistochemical (**D**) kidney injury molecule-1 (KIM-1) and (**E**) iron staining on day 7 in renal sections of Hmox1^lox/lox^ and Hmox1^R26Δ/Δ^ sham mice and Stx-challenged Hmox1^lox/lox^ and Hmox1^R26Δ/Δ^ mice (*n* = 5 per group). Arrowheads indicate (**C**) tubular dilatation and loss of the brush border in proximal tubules, (**D**) KIM-1-positive tubules. Scale bars = 100 µm. Data are expressed as (**A**–**E**) scatter dot plots with median (interquartile range) for n observations. * *p* < 0.05 vs. corresponding sham group, ^§^ *p* < 0.05 vs. Hmox1^lox/lox^ Stx group (Mann–Whitney *U* test).

The plasma levels of neutrophil gelatinase-associated lipocalin (NGAL, Figure 3A) and urea (Figure 3B) were elevated in Stx-challenged Hmox1^lox/lox^ and Hmox1^R26Δ/Δ^ mice compared to their corresponding sham group, indicating kidney injury. NGAL plasma levels were 1.7-fold higher (*p* < 0.0001) in Stx-challenged Hmox1^R26Δ/Δ^ compared to Stx-challenged Hmox1^lox/lox^ mice (Figure 3A). Urea plasma levels showed a statistically nonsignificant 1.3-fold (*p* = 0.06) increase in Stx-challenged Hmox1^R26Δ/Δ^ compared to Stx-challenged Hmox1^lox/lox^ mice (*p* = 0.06) (Figure 3B). Renal morphology evaluated by periodic acid Schiff (PAS) score was altered in Stx-challenged Hmox1^lox/lox^ and Hmox1^R26Δ/Δ^ mice compared to their corresponding sham group (Figure 3C). Similarly, kidney injury molecule-1 (KIM-1) expression was increased in Stx-challenged Hmox1^lox/lox^ and Hmox1^R26Δ/Δ^ mice compared to their corresponding sham group (Figure 3D). No renal iron depositions were detected in Hmox1^lox/lox^ and Hmox1^R26Δ/Δ^ mice irrespective of Stx challenge (Figure 3E).

### 2.4. Effect of Hmox1 Deficiency on Renal Thrombotic Microangiopathy in Mice with HUS

**Figure 4:**
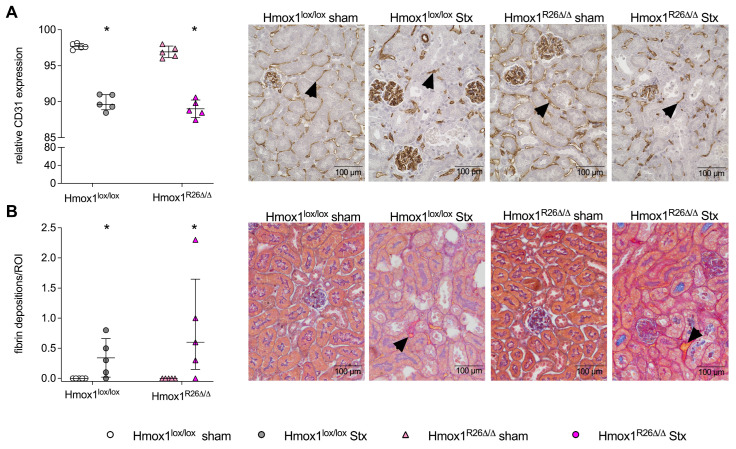
Renal thrombotic microangiopathy in Hmox1^lox/lox^ and Hmox1^R26Δ/Δ^ mice with experimental hemolytic-uremic syndrome (HUS). Quantification and representative pictures of (**A**) CD31 and (**B**) acid fuchsin orange G (SFOG) staining on day 7 in renal sections of Hmox1^lox/lox^ and Hmox1^R26Δ/Δ^ sham mice and Stx-challenged Hmox1^lox/lox^ and Hmox1^R26Δ/Δ^ mice (*n* = 5 per group). Arrowheads indicate (**A**) CD31-positive endothelial cells and (**B**) SFOG-positive areas indicative for fibrin deposition. Scale bars = 100 µm. Data are expressed as scatter dot plots with median (interquartile range) for *n* observations. * *p* < 0.05 vs. corresponding sham group (Mann–Whitney *U* test).

Endothelial cell damage and the appearance of fibrin deposits are typical signs of TMA, a condition often observed in patients with HUS [25]. In the kidneys of Stx-challenged Hmox1^lox/lox^ and Hmox1^R26Δ/Δ^ mice, a markedly diminished expression of CD31 was noted in comparison to their corresponding sham group (*p* = 0.0079; Figure 4A) indicating a disturbance in the endothelial barrier after Stx-challenge. The presence of fibrin depositions, which serve as an indicator for microthrombus formation, was observed in renal sections of Stx-challenged Hmox1^lox/lox^ and Hmox1^R26Δ/Δ^ mice (*p* = 0.0476), but not in their corresponding sham mice (Figure 4B).

## 3. Discussion

In various inflammatory and hemolytic diseases HO-1 has been associated with tissue-protective effects, which are presumably mediated by the degradation of cytotoxic heme [26,27,28] or the production of potentially protective molecules, such as CO [29,30,31] and unconjugated bilirubin [32,33,34]. Nevertheless, the role of HO-1 in HUS has not yet been investigated in vivo in a systematic manner. Some evidence from in vitro experiments suggests that HO-1 may be involved in the pathology of HUS: Bitzan et al. demonstrated that HO-1 induction can ameliorate Stx-enhanced heme toxicity in renal epithelial cells [35], while Vareille et al. reported that HO-1 modulates nitric oxide (NO) production in response to STEC in human enterocytes [36]. It is noteworthy that Wijnsma et al. observed a positive correlation between HO-1 and the amount of free heme in the plasma of patients with HUS [24]. It remains unclear whether plasmatic HO-1 levels could be used as a prognostic marker. As recently demonstrated by our group, HO-1 is induced in the kidneys of Stx-challenged mice with moderate hemolysis [23]. These findings suggest a potential relevance of HO-1 in HUS, although its precise function remains to be elucidated.

The objective of this study was to investigate the impact of induction of HO-1 on the pathogenesis and progression of HUS by investigating the effects of *Hmox1* deficiency on clinical outcome, kidney injury and TMA in a mouse model of HUS-like disease. To this end, we subjected Hmox1^R26Δ/Δ^ mice with TMX-inducible HO-1 deficiency and Hmox1^lox/lox^ control mice to repetitive Stx administration. This mouse model goes back to Sauter et al., who administered Stx by intraperitoneal injection [37]. We have recently modified the model of Stx-induced HUS by repeated intravenous administering of Stx [38]. Given our hypothesis that altered HO-1 expression would have the greatest impact on Stx-induced pathological findings in the kidneys, we initially examined the effect of Cre/LoxP-mediated reduction of *Hmox1* on renal tissue. Stx exposure resulted in increased renal HO-1 levels in Hmox1^R26Δ/Δ^ and Hmox1^lox/lox^ mice, indicating that *Hmox1* gene expression in the kidneys of Hmox1^R26Δ/Δ^ mice is not entirely suppressed and that residual *Hmox1* mRNA is sufficient for an induction of renal HO-1. However, the Stx-induced HO-1 expression was markedly diminished in the Hmox1^R26Δ/Δ^ mice compared to the Hmox1^lox/lox^ mice. Complete gene deletion is often challenging, as recombination efficacy varies depending on the tissue type [39,40]. It should be noted that this is a limitation of the model system that we employed. However, Bolisetty et al. demonstrated that a conditional knockout of *Hmox1* in renal tissue in mice is sufficient to demonstrate the influence of HO-1 induction on disease progression [41]. The authors demonstrated an exacerbation of renal disease in a cisplatin model of AKI with local deletion of HO-1 in proximal tubular cells [41]. As an alternative, constitutive homozygous *Hmox1* knockout mice are not necessarily the optimal choice, as they have been described to have a high pre- and postnatal mortality rate [42,43,44,45]. It is proposed that the small number of homozygous *Hmox1* knockout mice that survived may have developed compensatory mechanisms that remain unknown. In contrast, no phenotypic and clinical anomalies were observed in in *Hmox1*^R26Δ/Δ^ compared to *Hmox1*^lox/lox^ mice prior to the start of the experiment at day 0.

The *Hmox1*^R26Δ/Δ^ mice, which exhibited a reduction in HO-1 expression in the kidneys relative to the *Hmox1*^lox/lox^ mice, demonstrated a notable elevation in plasma NGAL levels and a marginal increase in plasma urea levels upon Stx challenge, when compared to the Hmox1^lox/lox^ mice. NGAL is a well characterized biomarker for tubular damage and is released by the distal tube in states of acute kidney injury involving the tubules [46,47]. Some studies even suggest that plasma NGAL levels may be superior to serum creatinine concentrations and KIM-1 for the prediction of development of acute kidney injury [47,48]. Interestingly, it has been shown that inflammatory events such as infections of the liver [49], lungs [50] and intestine [51] also lead to an increase in NGAL in plasma. However, previous studies of the liver, lung and colon in the mouse model used in this study showed a slight infiltration of inflammatory cells but no significant tissue damage to these organs [23]. The increase in plasma NGAL and urea levels in *Hmox1*^R26Δ/Δ^ mice with experimental HUS may therefore indicate that HO-1 could be involved in maintaining kidney function and protecting against Stx-mediated kidney damage. Furthermore, clinical symptoms of disease, as indicated by the HUS score, occurred two days earlier in *Hmox1*^R26Δ/Δ^ mice than in *Hmox1*^lox/lox^ mice with HUS. Nevertheless, the reduction in renal HO-1 in *Hmox1*^R26Δ/Δ^ mice compared to *Hmox1*^lox/lox^ mice with HUS at day 7 did not lead to differences in survival, weight loss or disease progression in our experimental setup. Furthermore, other markers of kidney injury, including morphological changes, KIM-1 expression, vascular fibrin depositions and endothelial cell loss, were not different between Hmox1^lox/lox^ and Hmox1^R26Δ/Δ^ mice with HUS. In contrast, Larsen et al. reported in a murine model of polymicrobial sepsis that *Hmox1* deficiency exacerbates tissue damage leading to the development of severe sepsis and host death [8]. Furthermore, in a murine model of cerebral malaria [52] and non-cerebral malaria [21], it has been demonstrated that *Hmox1* deficiency exacerbates disease progression, resulting in host death irrespective of parasitemia. Conversely, *Hmox1* overexpression has been shown to suppress the pathogenesis of these diseases [21,52]. In a murine model of cyclosporine A-induced kidney injury, *Hmox1* deficiency was associated with an increased mortality rate, renal fibrosis, inflammation (as indicated by elevated levels of the cytokine interleukin-6) and apoptosis [53].

In light of the residual HO-1 levels observed in the kidneys of Hmox1^R26Δ/Δ^ mice in this study, it is pertinent to consider the findings in the existing scientific literature, which raises the question of whether a constitutive *Hmox1* knockout might have resulted in further differences in kidney damage and disease progression in mice with HUS, as has been observed in other disease contexts [8,11,21,52,53].

The presence of renal [54] and hepatic [55,56] iron depositions has been documented in case reports of patients with *Hmox1* deficiency and in renal tissue of HO-1-deficient mice with a *Hmox1* knockout [57,58,59]. These findings align with our previously reported observation of iron depositions in constitutive haptoglobin knockout mice [23], which is another example for a preclinical model with a disturbed iron homeostasis [60]. In this study, we did not detect renal iron depositions in Hmox1^R26Δ/Δ^ mice with or without HUS, thereby suggesting that residual HO-1 levels in the kidneys of these mice are sufficient to maintain iron homeostasis.

In the context of HUS pathology, the prevailing hypothesis posits that hemolysis is primarily mediated by the development of renal TMA [4,61]. We previously reported that repetitive Stx challenge in mice resulted in the development of renal TMA and hemolysis [23,38]. In Hmox1^lox/lox^ as well as Hmox1^R26Δ/Δ^ mice, Stx challenge was associated with comparable loss of endothelial cells and fibrin depositions in the kidneys, which can be considered as surrogate parameters for the development of renal TMA. This observation suggests that renal HO-1 induction is unlikely to be a critical factor for the development of renal TMA in our murine model of Stx-induced HUS-like disease.

## 4. Conclusions

In conclusion, the results of this study suggest that HO-1 might play a protective role in the pathogenesis of Stx-induced HUS. The attenuation of renal HO-1 induction of about 43% was found to be sufficient to increase NGAL, a surrogate parameter for kidney injury, by about 1.7-fold in the plasma of Hmox1^R26Δ/Δ^ mice compared to Hmox1^lox/lox^ with HUS. However, the clinical presentation of mice (monitored by the HUS score), weight loss and other markers of renal injury (e.g., KIM-1, PAS score) were not significantly affected by the reduction in renal HO-1. To gain further insight into the role of HO-1 in HUS with regard to clinical outcome and histopathological changes, further studies with a prolonged observation period or a constitutive *Hmox1* knockout might be considered.

## 5. Materials and Methods

### 5.1. Mice with Tamoxifen-Induced Hmox1 Gene Deletion and Corresponding Control Mice

Constitutive homozygous *Hmox1* knockout mice have been described to have a high embryonic mortality rate [42,43]. Therefore, we used C57BL/6J-R26^cre/cre ERT2^Hmox1^lox/lox^ (Hmox1^R26Δ/Δ^) mice and C57BL/6J-R26^wt/wt ERT2^Hmox1^lox/lox^ (Hmox1^lox/lox^) mice for the experiments, which were kindly provided by Dr. Miguel P. Soares (Instituto Gulbenkian de Ciência, Oeiras, Portugal) [62]. Conditional gene deletion of *Hmox1* was induced in 8 week-old male mice by feeding TMX supplemented diet for 6 weeks (M-Z, Low phytoestrogen; 360 mg/kg TAM citrate + sucrose flavor, 10 mm soybean free diet, sterilized 25 kGy; SSNIFF Spezialdiäten, Soest, Germany). Sample size was determined a priori using the G*Power 3.1.9.7 software as described by Faul et al. [63]. Hmox1^R26Δ/Δ^ and Hmox1^lox/lox^ were housed in separate cages with up to 5 animals per cage and a 14 h/10 h day/night cycle. Cages were equipped with wire bar grid for standard rodent food, water supplied ad libitum and cages as well as enrichment (nesting material, tunnel) cleaned once per week. Conditions were maintained at 21 °C ± 2 °C with a relative humidity of 55% ± 10% and each cage was ventilated individually. Sample collection was distributed over 3 independent animal experiments (*n* = 6, *n* = 23, *n* = 26). One animal in the Hmox1^lox/lox^ group was euthanized and therefore excluded from further analyses due to circulatory collapse during first experimental HUS induction. Animal experiments were approved by the regional animal welfare committee (Thuringian State Office for Food Safety and Consumer Protection, Bad Langensalza, Germany, registration number 02-040/16) and performed in accordance with German legislation as well as the ARRIVE guidelines.

### 5.2. Stx2 Purification

Fast protein liquid chromatography was used for purification of Stx2 produced from an O157:H7 enterohemorrhagic *Escherichia coli* strain 86–24 patient isolate [38] that exclusively produces Stx2 [64]. LC_50_ (concentration of Stx2 which is lethal to 50% of the cells) was calculated with Vero cells using a neutral red assay (TOX4, Merck KGaA, Darmstadt, Germany) and determined as 9.51 pg/mL.

### 5.3. Hmox1 Gene Expression and Analysis

To investigate *Hmox1* expression, RNA was isolated on the last day of TMX diet from 7–45 µL murine blood obtained from the vena facialis using the NucleoSpin^®^ RNA Blood Kit on a C1000^TM^ Thermal Cycler (Bio-Rad Laboratories Inc., Hercules, CA, USA). Transcription of 100 ng RNA into complementary DNA was performed on a C1000^TM^ Thermal Cycler (Bio-Rad Laboratories Inc.) using the iScript^TM^ Reverse Transcription Supermix for RT-qPCR Kit. Gene expression analysis of 50 ng cDNA was performed in duplicates by quantitative real-time PCR on a CFX96 Touch Real-Time PCR Detection System (Bio-Rad Laboratories, Inc.) using iTaq Universal SsoAdvanced SYBR^®^ Green Supermix (Bio-Rad Laboratories, Inc.) and PrimePCR™ assays according to the manufacturer’s protocol. Commercial kits are listed in Supplementary Table S1. Primer sequences for *Hmox1* and the reference gene *acidic ribosomal phosphoprotein P0* (*mArbP0*) are listed in Supplementary Table S2. *Hmox1* levels were normalized to the reference gene and relative changes in comparison to the control group calculated utilizing the ∆∆Cq method [65] implemented in the CFX Manager 3.1.3090.1022 software (Bio-Rad Laboratories, Inc.). In accordance with the software, if both measured values were below the detection limit, the estimated expression was set to zero. If one of the replicates did not reach a fluorescence level above the quantification threshold, the relative expression was calculated using the other one.

### 5.4. Experimental HUS

Experimental HUS was induced in Hmox1^lox/lox^ and Hmox1^R26Δ/Δ^ mice 2 weeks after termination of TMX feeding (housing conditions see Section 5.1). Animals were randomly allocated to control (Hmox1^lox/lox^ sham (*n* = 12), Hmox1^R26Δ/Δ^ sham (*n* = 15)) and experimental groups (Hmox1^lox/lox^ Stx (*n* = 12), Hmox1^R26Δ/Δ^ Stx (*n* = 15)). Mice were subjected to 25 ng/kg bodyweight (BW) Stx2 (Stx groups) or vehicle (0.9% NaCl, sham groups) i.v. on days 0, 3 and 6 accompanied by fluid resuscitation with 800 µL Ringer’s lactate solution s.c. three times daily to prevent dehydration [38]. To monitor disease progression, BW was measured every 24 h and HUS scores (for grading and score calculation see Supplementary Table S3) were determined three times daily. Mice were euthanized in deep ketamine/xylazine anesthesia (100 mg/kg BW ketamine, 10 mg/kg BW xylazine) on day 7 or upon reaching humane endpoints to comply with ethical regulations (see termination criteria in Supplementary Table S3). Blood was taken from the vena cava and remaining blood was removed by perfusing the animal with 0.9% NaCl before harvesting the organs. The outcome measures for each mouse were obtained in a blinded manner. Solutions and drugs are listed in Supplementary Table S4.

### 5.5. Western Blot Analysis

For Western blot analysis *n* = 12 samples per group of the Hmox1^lox/lox^ strain and *n* = 15 samples per group of the Hmox1^R26Δ/Δ^ strain were used. For blotting of renal HO-1 20–30 mg kidney tissue was homogenized in ice-cold lysis buffer (10 µL/1 mg tissue, recipe in Supplementary Table S5). After 15 min on ice the homogenate was centrifuged (7 min, 4 °C, 900× *g*). Protein concentration of the supernatant was assessed spectrophotometrically (Spark^®^ multimode microplate reader, Tecan Group Ltd., Männedorf, Switzerland) using the Pierce Detergent Compatible Bradford Assay Kit at 595 nm. Commercial kits are listed in Supplementary Table S1.

Comparisons of interest were investigated by analyzing individual samples of the corresponding groups on the same membrane (Figure 1B–E; Supplementary Figure S1A–D). In Figure 1B–E and Supplementary Figure S1A–D the same mouse samples per group were used for each comparison. Per lane and sample, 50 µg of whole tissue protein was added (Mini-Protean^®^ Tetra cell vertical electrophoresis system, Bio-Rad Laboratories, Inc.). For an overview gel *n* = 12 samples per group of the Hmox1^lox/lox^ strain and *n* = 15 samples per group of the Hmox1^R26Δ/Δ^ strain were pooled to equal protein amounts (Figure 1F). A total of 100 µg protein of the pooled samples per group was used for the overview gel (Figure 1F; Electrophoresis Unit ROTIPHORESE^®^ PROclamp MINI Wide, Carl Roth GmbH + Co. KG). Proteins of all samples were separated on 1 mm thick hand-cast gels (TGX Stain-Free™ FastCast™ Acrylamide Kit, 10%) using a running buffer (recipe in Supplementary Table S5) and applying constant 100 V for approx. 10 min, and subsequently constant 40 mA until the dye front ran off the bottom of the gel. The proteins were transferred to methanol-activated low fluorescent polyvinylidene difluoride membranes (Bio-Rad Laboratories Inc.) at constant 25 V for 5 min using the Trans-Blot Turbo RTA Transfer Kit and Trans-Blot Turbo Transfer system (Bio-Rad Laboratories Inc.). Membranes were blocked for 2 h at room temperature in 5% bovine serum albumin (BSA, 11930.04, SERVA Electrophoresis GmbH, Heidelberg, Germany) dissolved in TRIS-buffered saline-Tween buffer (TBS-T, recipe in Supplementary Table S5). Antibodies and incubation conditions are provided in Supplementary Table S6. Washing steps were performed 3 times for 5 min with TBS-T at room temperature. For signal detection, Clarity Max Western ECL substrate and the ChemiDoc MP Imaging System (Bio-Rad Laboratories Inc.) were used. Signals were analyzed using the Image Lab software 6.1.0.07 (Bio-Rad Laboratories Inc.). Levels of proteins of interest were normalized to the total protein signal across the whole membrane using the stain-free technology (StainFree, Bio-Rad Laboratories Inc.) [66].

### 5.6. Blood and Plasma Sample Analysis

To obtain plasma, heparin (Heparin-Natrium Braun 25.000 I.E./5 mL injection solution, B. Braun SE, Melsungen, Germany) anti-coagulated blood was centrifuged (10 min, 4 °C, 3000× *g*). NGAL and urea levels were analyzed with commercial kits according to the manufacturer’s instructions (kits in Supplementary Table S1) on the Spark^®^ multimode microplate reader (Tecan Group Ltd., Männedorf, Switzerland). Samples were measured as technical replicates (SparkControl^TM^, Version 2.1) and the median calculated as appropriate. Samples with absorption values outside of the standard curve were excluded from statistical analyses. Due to technical reasons, this affected one sample of the Hmox1^lox/lox^ sham group and the Hmox1^R26Δ/Δ^ sham group in the analysis of NGAL and one sample from the Hmox1^lox/lox^ Stx group in the analysis of urea. The concentration was calculated with GraphPad Prism 10.2 (GraphPad Software, Inc., Boston, MA, USA) and the data further prepared for statistics with R 4.3.2 [67].

### 5.7. Tissue Preparation and Histological and Immunohistochemical Analysis

For histological and immunohistochemical stainings, a cohort subset of five mice per group were chosen according to their plasma urea level (closest to the mean value). After removal, kidneys were fixed immediately, processed and embedded in paraffin as described previously [38]. Deparaffinization, hydration of 2 µm renal sections and staining for PAS, KIM-1, CD31 and acid fuchsin orange G (SFOG) were performed as described previously [38]. KIM-1 and CD31 antibodies are listed in Supplementary Table S7. Evaluation and quantification of histomorphological changes (PAS) and kidney injury (KIM-1 expression) were performed as described previously [38]. Quantification of endothelial cells (CD31 staining) was performed by using a 40× objective and an ocular with integrated grid (with 10 × 10 fields) which was superimposed on 20 cortical areas (adjacent to one another) of each section. The number of fields with positive (brown) staining per grid was counted and the mean value of 20 grids per section was used for analysis [38]. Signal strength was not taken into account for the quantification, as the staining intensity may vary due to technical factors. Quantification of SFOG staining (detection of thrombus formation) was carried out using a 40× objective and by counting the number of fibrin depositions per field (ROI) in 10 cortical fields per section. For further analysis the mean value of 10 ROIs per section was used [23,38]. Performance and quantification of iron staining by Berliner-Blau (HEMATOGNOSE FE^®^) were carried out as described previously [23]. Final values for further analysis are the mean values of all analyzed fields or grid areas for each animal of each parameter. Representative images of PAS, KIM-1 and CD31 staining were acquired using a KEYENCE BZ-X800 microscope and the BZ-X800 Viewer software Version 1.1.1 (KEYENCE Deutschland GmbH, Neu-Isenburg, Germany) after auto exposure and adjusting with white balance at a magnification of 40×. Representative images of SFOG and iron staining were acquired using an Olympus Bx60 microscope with a XC30 camera and cellSens software version 1.9 (Olympus Deutschland, Hamburg, Germany) after auto exposure and adjusting with white balance at a magnification of 20×.

### 5.8. Statistics

G*Power 3.1.9.2 was used to determine the required group size a priori [63]. An alpha error of 0.05, power (1-beta) of 0.80 and a Cohen’s effect size of 0.4 were set as basis and one-way ANOVA used for the a priori power analysis. Data were analyzed with GraphPad Prism 10.2 and are depicted as median with interquartile range for *n* observations (see figure legends). To evaluate statistically significant differences, the Mann–Whitney *U* test was used to test the following comparisons: Hmox1^lox/lox^ to the Hmox1^R26Δ/Δ^ prior to HUS induction; Stx group to the corresponding sham group of each strain respectively; the sham group of one strain to the sham group of the other strain as well as the Stx groups of each strain to each other. A *p*-value < 0.05 was considered significant.

## Data Availability

The original contributions presented in the study are included in the article/Supplementary Material, further inquiries can be directed to the corresponding authors.

## Supplementary Materials

The following supporting information can be downloaded at: https://www.mdpi.com/article/10.3390/toxins16120543/s1, Supplementary Figure S1A–D: Immunoblots for Figure 1B–E, Supplementary Table S1: Commercial kits, Supplementary Table S2: Primer sequences for detection of *Hmox1* gene expression, Supplementary Table S3: HUS score, Supplementary Table S4: Solutions and drugs used for experimental HUS, Supplementary Table S5: Buffers used for Western blot, Supplementary Table S6: Primary and secondary antibodies used for immunoblot analyses, Supplementary Table S7: Primary and secondary antibodies used for immunohistochemistry.

## References

[1] Fruth A., Lang C., Größl T., Garn T., Flieger A. (2024). Genomic surveillance of STEC/EHEC infections in Germany 2020 to 2022 permits insight into virulence gene profiles and novel O-antigen gene clusters. Int. J. Med. Microbiol..

[2] Rivas M., Pichel M., Colonna M., Casanello A.L., Alconcher L.F., Galavotti J., Principi I., Araujo S.P., Ramírez F.B., González G. (2023). Surveillance of Shiga toxin-producing Escherichia coli associated bloody diarrhea in Argentina. Rev. Argent. Microbiol..

[3] Seliga-Gąsior D., Sokól-Leszczyñska B., Krzysztoñ-Russjan J., Wierzbicka D., Stępieñ-Hołubczat K., Lewandowska P., Frankiewicz E., Cacko A., Leszczyñska B., Demkow U. (2024). Epidemiological Characteristics of Shiga Toxin-Producing Escherichia coli Responsible for Infections in the Polish Pediatric Population. Pol. J. Microbiol..

[4] Fakhouri F., Zuber J., Frémeaux-Bacchi V., Loirat C. (2017). Haemolytic uraemic syndrome. Lancet.

[5] Ruggenenti P., Noris M., Remuzzi G. (2001). Thrombotic microangiopathy, hemolytic uremic syndrome, and thrombotic thrombocytopenic purpura. Kidney Int..

[6] Liu Y., Thaker H., Wang C., Xu Z., Dong M. (2022). Diagnosis and Treatment for Shiga Toxin-Producing Escherichia coli Associated Hemolytic Uremic Syndrome. Toxins.

[7] Alconcher L.F., Lucarelli L.I., Bronfen S. (2023). Long-term kidney outcomes in non-dialyzed children with Shiga-toxin Escherichia coli associated hemolytic uremic syndrome. Pediatr. Nephrol..

[8] Larsen R., Gozzelino R., Jeney V., Tokaji L., Bozza F.A., Japiassú A.M., Bonaparte D., Cavalcante M.M., Chora A., Ferreira A. (2010). A central role for free heme in the pathogenesis of severe sepsis. Sci. Transl. Med..

[9] Merle N.S., Grunenwald A., Rajaratnam H., Gnemmi V., Frimat M., Figueres M.L., Knockaert S., Bouzekri S., Charue D., Noe R. (2018). Intravascular hemolysis activates complement via cell-free heme and heme-loaded microvesicles. JCI Insight.

[10] Elphinstone R.E., Conroy A.L., Hawkes M., Hermann L., Namasopo S., Warren H.S., John C.C., Liles W.C., Kain K.C. (2016). Alterations in Systemic Extracellular Heme and Hemopexin Are Associated With Adverse Clinical Outcomes in Ugandan Children With Severe Malaria. J. Infect. Dis..

[11] Liu Y., Jing F., Yi W., Mendelson A., Shi P., Walsh R., Friedman D.F., Minniti C., Manwani D., Chou S.T. (2018). HO-1(hi) patrolling monocytes protect against vaso-occlusion in sickle cell disease. Blood.

[12] Belcher J.D., Mahaseth H., Welch T.E., Otterbein L.E., Hebbel R.P., Vercellotti G.M. (2006). Heme oxygenase-1 is a modulator of inflammation and vaso-occlusion in transgenic sickle mice. J. Clin. Investig..

[13] Vissa M., Larkin S.K., Vichinsky E.P., Kuypers F.A., Soupene E. (2023). Assessment of total and unbound cell-free heme in plasma of patients with sickle cell disease. Exp. Biol. Med..

[14] Gbotosho O.T., Kapetanaki M.G., Kato G.J. (2020). The Worst Things in Life are Free: The Role of Free Heme in Sickle Cell Disease. Front. Immunol..

[15] Campbell N.K., Fitzgerald H.K., Dunne A. (2021). Regulation of inflammation by the antioxidant haem oxygenase 1. Nat. Rev. Immunol..

[16] Ryter S.W., Alam J., Choi A.M. (2006). Heme oxygenase-1/carbon monoxide: From basic science to therapeutic applications. Physiol. Rev..

[17] Gozzelino R., Jeney V., Soares M.P. (2010). Mechanisms of cell protection by heme oxygenase-1. Annu. Rev. Pharmacol. Toxicol..

[18] Ryter S.W. (2022). Heme Oxygenase-1: An Anti-Inflammatory Effector in Cardiovascular, Lung, and Related Metabolic Disorders. Antioxidants.

[19] Dutra F.F., Bozza M.T. (2014). Heme on innate immunity and inflammation. Front. Pharmacol..

[20] Vinchi F., Costa da Silva M., Ingoglia G., Petrillo S., Brinkman N., Zuercher A., Cerwenka A., Tolosano E., Muckenthaler M.U. (2016). Hemopexin therapy reverts heme-induced proinflammatory phenotypic switching of macrophages in a mouse model of sickle cell disease. Blood.

[21] Seixas E., Gozzelino R., Chora A., Ferreira A., Silva G., Larsen R., Rebelo S., Penido C., Smith N.R., Coutinho A. (2009). Heme oxygenase-1 affords protection against noncerebral forms of severe malaria. Proc. Natl. Acad. Sci. USA.

[22] de Azevedo-Quintanilha I.G., Medeiros-de-Moraes I.M., Ferreira A.C., Reis P.A., Vieira-de-Abreu A., Campbell R.A., Weyrich A.S., Bozza P.T., Zimmerman G.A., Castro-Faria-Neto H.C. (2020). Haem oxygenase protects against thrombocytopaenia and malaria-associated lung injury. Malar. J..

[23] Pirschel W., Mestekemper A.N., Wissuwa B., Krieg N., Kröller S., Daniel C., Gunzer F., Tolosano E., Bauer M., Amann K. (2022). Divergent roles of haptoglobin and hemopexin deficiency for disease progression of Shiga-toxin-induced hemolytic-uremic syndrome in mice. Kidney Int..

[24] Wijnsma K.L., Veissi S.T., de Wijs S., van der Velden T., Volokhina E.B., Wagener F., van de Kar N., van den Heuvel L.P. (2020). Heme as Possible Contributing Factor in the Evolvement of Shiga-Toxin Escherichia coli Induced Hemolytic-Uremic Syndrome. Front. Immunol..

[25] Lusco M.A., Fogo A.B., Najafian B., Alpers C.E. (2016). AJKD Atlas of Renal Pathology: Thrombotic Microangiopathy. Am. J. Kidney Dis..

[26] Grinshtein N., Bamm V.V., Tsemakhovich V.A., Shaklai N. (2003). Mechanism of low-density lipoprotein oxidation by hemoglobin-derived iron. Biochemistry.

[27] Graça-Souza A.V., Arruda M.A., de Freitas M.S., Barja-Fidalgo C., Oliveira P.L. (2002). Neutrophil activation by heme: Implications for inflammatory processes. Blood.

[28] Wagener F.A., Eggert A., Boerman O.C., Oyen W.J., Verhofstad A., Abraham N.G., Adema G., van Kooyk Y., de Witte T., Figdor C.G. (2001). Heme is a potent inducer of inflammation in mice and is counteracted by heme oxygenase. Blood.

[29] Kaide J.I., Zhang F., Wei Y., Jiang H., Yu C., Wang W.H., Balazy M., Abraham N.G., Nasjletti A. (2001). Carbon monoxide of vascular origin attenuates the sensitivity of renal arterial vessels to vasoconstrictors. J. Clin. Investig..

[30] Brouard S., Otterbein L.E., Anrather J., Tobiasch E., Bach F.H., Choi A.M., Soares M.P. (2000). Carbon monoxide generated by heme oxygenase 1 suppresses endothelial cell apoptosis. J. Exp. Med..

[31] Ryter S.W. (2021). Significance of Heme and Heme Degradation in the Pathogenesis of Acute Lung and Inflammatory Disorders. Int. J. Mol. Sci..

[32] Sedlak T.W., Saleh M., Higginson D.S., Paul B.D., Juluri K.R., Snyder S.H. (2009). Bilirubin and glutathione have complementary antioxidant and cytoprotective roles. Proc. Natl. Acad. Sci. USA.

[33] Manikanta, NaveenKumar S.K., Thushara R.M., Hemshekhar M., Sumedini M.L., Sunitha K., Kemparaju K., Girish K.S. (2024). Counteraction of unconjugated bilirubin against heme-induced toxicity in platelets. Thromb. Res..

[34] Huang S.S., Ding Y., Yi X.N., Mao H.Y., Xie Z.Y., Shen X.K., Lu Y., Yan J., Wang Y.W., Yang Z.X. (2024). Exploring the inverse relationship between serum total bilirubin and systemic immune-inflammation index: Insights from NHANES data (2009–2018). Eur. J. Med. Res..

[35] Bitzan M., Bickford B.B., Foster G.H. (2004). Verotoxin (shiga toxin) sensitizes renal epithelial cells to increased heme toxicity: Possible implications for the hemolytic uremic syndrome. J. Am. Soc. Nephrol..

[36] Vareille M., Rannou F., Thélier N., Glasser A.L., de Sablet T., Martin C., Gobert A.P. (2008). Heme oxygenase-1 is a critical regulator of nitric oxide production in enterohemorrhagic Escherichia coli-infected human enterocytes. J. Immunol..

[37] Sauter K.A., Melton-Celsa A.R., Larkin K., Troxell M.L., O’Brien A.D., Magun B.E. (2008). Mouse model of hemolytic-uremic syndrome caused by endotoxin-free Shiga toxin 2 (Stx2) and protection from lethal outcome by anti-Stx2 antibody. Infect. Immun..

[38] Dennhardt S., Pirschel W., Wissuwa B., Daniel C., Gunzer F., Lindig S., Medyukhina A., Kiehntopf M., Rudolph W.W., Zipfel P.F. (2018). Modeling Hemolytic-Uremic Syndrome: In-Depth Characterization of Distinct Murine Models Reflecting Different Features of Human Disease. Front. Immunol..

[39] Kumar R., Mao Y., Patial S., Saini Y. (2022). Induction of whole-body gene deletion via R26-regulated tamoxifen-inducible Cre recombinase activity. Front. Pharmacol..

[40] Ilchuk L.A., Stavskaya N.I., Varlamova E.A., Khamidullina A.I., Tatarskiy V.V., Mogila V.A., Kolbutova K.B., Bogdan S.A., Sheremetov A.M., Baulin A.N. (2022). Limitations of Tamoxifen Application for In Vivo Genome Editing Using Cre/ER(T2) System. Int. J. Mol. Sci..

[41] Bolisetty S., Traylor A., Joseph R., Zarjou A., Agarwal A. (2016). Proximal tubule-targeted heme oxygenase-1 in cisplatin-induced acute kidney injury. Am. J. Physiol. Renal Physiol..

[42] Zelenay S., Chora A., Soares M.P., Demengeot J. (2006). Heme oxygenase-1 is not required for mouse regulatory T cell development and function. Int. Immunol..

[43] Yoshida T., Maulik N., Ho Y.S., Alam J., Das D.K. (2001). H(mox-1) constitutes an adaptive response to effect antioxidant cardioprotection: A study with transgenic mice heterozygous for targeted disruption of the Heme oxygenase-1 gene. Circulation.

[44] Rana M., Choubey P., Nandi G., Jain S., Bajaj D., Sharma S., Basu-Modak S. (2023). Expression of angiogenic factors in the placenta of heme oxygenase-1 deficient mouse embryo. Reprod. Biol..

[45] Meyer N., Langwisch S., Scharm M., Zenclussen A.C. (2020). Using ultrasound to define the time point of intrauterine growth retardation in a mouse model of heme oxygenase-1 deficiency†. Biol. Reprod..

[46] Marakala V. (2022). Neutrophil gelatinase-associated lipocalin (NGAL) in kidney injury—A systematic review. Clin. Chim. Acta.

[47] Pan H.C., Yang S.Y., Chiou T.T., Shiao C.C., Wu C.H., Huang C.T., Wang T.J., Chen J.Y., Liao H.W., Chen S.Y. (2022). Comparative accuracy of biomarkers for the prediction of hospital-acquired acute kidney injury: A systematic review and meta-analysis. Crit. Care.

[48] Haase-Fielitz A., Bellomo R., Devarajan P., Story D., Matalanis G., Dragun D., Haase M. (2009). Novel and conventional serum biomarkers predicting acute kidney injury in adult cardiac surgery--a prospective cohort study. Crit. Care Med..

[49] Xu M.J., Feng D., Wu H., Wang H., Chan Y., Kolls J., Borregaard N., Porse B., Berger T., Mak T.W. (2015). Liver is the major source of elevated serum lipocalin-2 levels after bacterial infection or partial hepatectomy: A critical role for IL-6/STAT3. Hepatology.

[50] Cowland J.B., Sørensen O.E., Sehested M., Borregaard N. (2003). Neutrophil gelatinase-associated lipocalin is up-regulated in human epithelial cells by IL-1 beta, but not by TNF-alpha. J. Immunol..

[51] Yoo D.Y., Ko S.H., Jung J., Kim Y.J., Kim J.S., Kim J.M. (2013). Bacteroides fragilis enterotoxin upregulates lipocalin-2 expression in intestinal epithelial cells. Lab. Investig..

[52] Pamplona A., Ferreira A., Balla J., Jeney V., Balla G., Epiphanio S., Chora A., Rodrigues C.D., Gregoire I.P., Cunha-Rodrigues M. (2007). Heme oxygenase-1 and carbon monoxide suppress the pathogenesis of experimental cerebral malaria. Nat. Med..

[53] Łoboda A., Mucha O., Podkalicka P., Sobczak M., Miksza-Cybulska A., Kaczara P., Jozkowicz A., Dulak J. (2018). Kidney injury by cyclosporine A is aggravated in heme oxygenase-1 deficient mice and involves regulation of microRNAs. Acta Biochim. Pol..

[54] Yachie A., Niida Y., Wada T., Igarashi N., Kaneda H., Toma T., Ohta K., Kasahara Y., Koizumi S. (1999). Oxidative stress causes enhanced endothelial cell injury in human heme oxygenase-1 deficiency. J. Clin. Investig..

[55] Tahghighi F., Parvaneh N., Ziaee V. (2019). Post-mortem Diagnosis of Heme Oxygenase-1 Deficiency by Whole Exome Sequencing in an Iranian Child. Int. J. Mol. Cell Med..

[56] Chau A.S., Cole B.L., Debley J.S., Nanda K., Rosen A.B.I., Bamshad M.J., Nickerson D.A., Torgerson T.R., Allenspach E.J. (2020). Heme oxygenase-1 deficiency presenting with interstitial lung disease and hemophagocytic flares. Pediatr. Rheumatol. Online J..

[57] Poss K.D., Tonegawa S. (1997). Heme oxygenase 1 is required for mammalian iron reutilization. Proc. Natl. Acad. Sci. USA.

[58] Bednarz A., Lipiński P., Starzyński R.R., Tomczyk M., Kraszewska I., Herman S., Kowalski K., Gruca E., Jończy A., Mazgaj R. (2020). Exacerbation of Neonatal Hemolysis and Impaired Renal Iron Handling in Heme Oxygenase 1-Deficient Mice. Int. J. Mol. Sci..

[59] Kopacz A., Klóska D., Cysewski D., Kraszewska I., Przepiórska K., Lenartowicz M., Łoboda A., Grochot-Przęczek A., Nowak W., Józkowicz A. (2023). Co-administration of angiotensin II and simvastatin triggers kidney injury upon heme oxygenase-1 deficiency. Free Radic. Biol. Med..

[60] Fagoonee S., Gburek J., Hirsch E., Marro S., Moestrup S.K., Laurberg J.M., Christensen E.I., Silengo L., Altruda F., Tolosano E. (2005). Plasma protein haptoglobin modulates renal iron loading. Am. J. Pathol..

[61] Mayer C.L., Leibowitz C.S., Kurosawa S., Stearns-Kurosawa D.J. (2012). Shiga toxins and the pathophysiology of hemolytic uremic syndrome in humans and animals. Toxins.

[62] DeSouza-Vieira T., Iniguez E., Serafim T.D., de Castro W., Karmakar S., Disotuar M.M., Cecilio P., Lacsina J.R., Meneses C., Nagata B.M. (2020). Heme Oxygenase-1 Induction by Blood-Feeding Arthropods Controls Skin Inflammation and Promotes Disease Tolerance. Cell Rep..

[63] Faul F., Erdfelder E., Lang A.G., Buchner A. (2007). G*Power 3: A flexible statistical power analysis program for the social, behavioral, and biomedical sciences. Behav. Res. Methods.

[64] Griffin P.M., Ostroff S.M., Tauxe R.V., Greene K.D., Wells J.G., Lewis J.H., Blake P.A. (1988). Illnesses associated with Escherichia coli O157:H7 infections. A broad clinical spectrum. Ann. Intern. Med..

[65] Livak K.J., Schmittgen T.D. (2001). Analysis of relative gene expression data using real-time quantitative PCR and the 2(-Delta Delta C(T)) Method. Methods.

[66] Gilda J.E., Gomes A.V. (2013). Stain-Free total protein staining is a superior loading control to β-actin for Western blots. Anal. Biochem..

[67] R Core Team (2023). R: A Language and Environment for Statistical Computing.

