# Supplementary material for: Reduction in Renal Heme Oxygenase-1 Is Associated with an Aggravation of Kidney Injury in Shiga Toxin-Induced Murine Hemolytic-Uremic Syndrome
Source: Toxins (Basel). 2024 Dec 14;16(12):543. doi: 10.3390/toxins16120543 (PMC11679022; doi:10.3390/toxins16120543)
Supplement: Supplementary file 1 [file toxins-16-00543-s001.zip › toxins-3280619-supplementary.pdf]

Reduction in Renal Heme Oxygenase-1 is Associated with an Aggravation of Kidney Injury in Shiga Toxin-Induced Murine Hemolytic-Uremic Syndrome

Antonio N. Mestekemper, Wiebke Pirschel, Nadine Krieg, Maria K. Paulmann, Christoph Daniel, Kerstin Amann and Sina M. Coldewey

Supplementary Material

Supplementary Figure S1......3

Supplementary Tables......4

Supplementary Table S1: Commercial kits. 4

Supplementary Table S2: Primer sequences for detection of *Hmox1* gene expression. 4

Supplementary Table S3: HUS score. 4

Supplementary Table S4: Solutions and drugs used for experimental HUS. 5

Supplementary Table S5: Buffers used for Western blot. 6

Supplementary Table S6: Primary and secondary antibodies used for immunoblot analyses. 7

Supplementary Table S7: Primary and secondary antibodies used for immunohistochemistry. 7

Supplementary Figure S1

Supplementary Figure S1A-D refers to Figures 1B-E in the main text.

Supplementary Figure S1A (refers to Figure 1B). Hmox1$^{lox/lox}$ sham (n = 12) vs. Hmox1$^{lox/lox}$ Stx (n = 12):

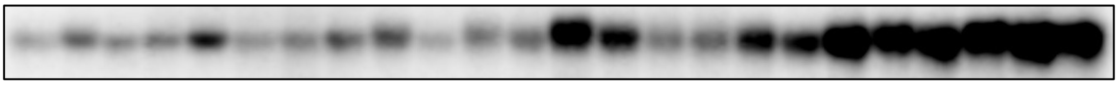

Supplementary Figure S1B (refers to Figure 1C). Hmox1$^{R26\Delta/\Delta}$ sham (n = 15) vs. Hmox1$^{R26\Delta/\Delta}$ Stx (n = 15):

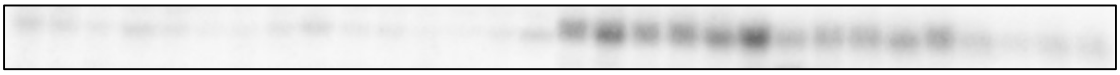

Supplementary Figure S1C (refers to Figure 1D). Hmox1$^{lox/lox}$ sham (n = 12) vs. Hmox1$^{R26\Delta/\Delta}$ sham (n = 15):

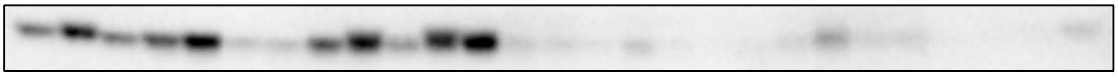

Supplementary Figure S1D (refers to Figure 1E). Hmox1$^{lox/lox}$ Stx (n = 12) vs. Hmox1$^{R26\Delta/\Delta}$ Stx (n = 15):

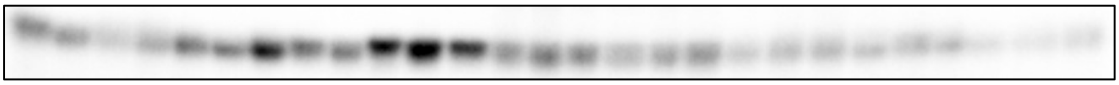

Supplementary Figure S1. Enlarged images from Figure 1B-E of the main text: HO-1 protein expression in the kidneys of Hmox1$^{lox/lox}$ and Hmox1$^{R26\Delta/\Delta}$ sham mice as well as Shiga-toxin (Stx)-challenged Hmox1$^{lox/lox}$ and Hmox1$^{R26\Delta/\Delta}$ mice at day 7 after initial HUS induction (Hmox1$^{lox/lox}$: n = 12 per group, Hmox1$^{R26\Delta/\Delta}$: n = 15 per group). (**A**) HO-1 expression in Hmox1$^{lox/lox}$ sham and Stx-challenged Hmox1$^{lox/lox}$ mice; (**B**) HO-1 expression of Hmox1$^{R26\Delta/\Delta}$ sham and Stx-challenged Hmox1$^{R26\Delta/\Delta}$ mice; (**C**) HO-1 expression of Hmox1$^{lox/lox}$ and Hmox1$^{R26\Delta/\Delta}$ sham mice; (**D**) HO-1 expression of Stx-challenged Hmox1$^{lox/lox}$ and Hmox1$^{R26\Delta/\Delta}$ mice.

Supplementary Tables

Supplementary Table S1: Commercial kits.

Target	Name	Cat. No.	Supplier
RNA isolation	NucleoSpin® RNA Blood Kit (50 preps)	740200.50	Macherey-Nagel, Düren, Germany
cDNA synthesis	iScriptTM Reverse Transcription Supermix for RT-qPCR Kit, 100 x 20 µL reactions	1708841	Bio-Rad Laboratories Inc., Feldkirchen, Germany
qPCR	iTaq Universal SsoAdvanced SYBR® Green Supermix, 200 x 20 µL reactions	1725120	Bio-Rad Laboratories Inc.
protein concentration	Pierce Detergent Compatible Bradford Assay Kit, 300 test sample tubes	23246	Thermo Fisher Scientific Inc., Darmstadt, Germany
1-D polyacrylamide gel electrophoresis for separation of proteins	TGX Stain-Free™ FastCast™ Acrylamide Kit, 10%	1610183	Bio-Rad Laboratories Inc.
protein transfer on membrane	Trans-Blot Turbo RTA Transfer Kit, 40 blots	1704272	Bio-Rad Laboratories Inc.
digital imaging	Clarity Western ECL substrate, 100 mL, with any HRP-conjugated secondary antibody	1705062	Bio-Rad Laboratories Inc.
plasma NGAL	LEGEND MAX™ Mouse NGAL (Lipocalin-2) ELISA Kit	443707	BioLegend, Inc., San Diego, USA
plasma urea	Urea Assay Kit	ab83362	Abcam Limited, Cambridge, UK

Cat. No. – catalogue number.

Supplementary Table S2: Primer sequences for detection of *Hmox1* gene expression.

Gene name	Species	Primer sequence (5′ – 3′)	Cat. No. and supplier
Hmox1	mouse	Fwd: TGACACCTGAGGTCAAGCAC	qMmuCID0040051 (Bio-Rad Laboratories Inc.)
		Rev: TCTCTGCAGGGGCAGTATCT	
mArbP0 (acidic ribosomal phosphoprotein P0), (reference gene)	mouse	Fwd: CTTTGGGCATCACCACGAA	biomers.net GmbH, Ulm, Germany
		Rev: GCTGGCTCCCACCTTGTCT	

Cat. No. – catalogue number ; Fwd – forward; Rev – reverse.

Supplementary Table S3: HUS score. The grade of disease was calculated from the sum of all points of all criteria. Grade 1 = no signs of illness (6 points); grade 2 = low-grade (7 - 10 points); grade 3 = mid-grade (11 - 18 points); grade 4 = high-grade (≥ 19 points); grade 5 = dead.

Point(s) per criterion	I. activity	II. reaction	III. posture	IV. general symptoms	V. neurological symptoms	VI. fur
1	active, strong	curious, fast movements	normal	none	none	shiny, even
2 for I, II, VI<br>*1 for III, IV, V*	lower activity, occasional interruptions	reduced attention, appropriate reaction	normal	none	none	blunt, adjacent
3	markedly reduced	reduced attention, delayed reaction	slightly hunched	loss of 10-20% BW in 48 h	beginning hind limb clasping	blunt, slightly ruffled
4	lethargic, no movements	none	strong hunch	loss of > 20% BW in 48 h	pronounced hind limb clasping	blunt, strong piloerection

Termination criteria: reaching a HUS score of 4 at two consecutive observation points, dehydration, dark discoloration of the lower abdomen or scrotum, seizures/apathy, weight loss >20% within 48 hours, difficulties with food and water intake, conspicuously reduced respiratory rate, gasping for breath.

BW – body weight.

Supplementary Table S4: Solutions and drugs used for experimental HUS.

Solution/drug	Product name	Supplier
0.9% NaCl	NaCl solution 0.9% ad us. vet.	WDT, Garbsen, Germany
Ringer`s lactate solution	Ringer`s lactate solution ad us. vet.	WDT
ketamine	Ketabel, 100 mg/mL	bela pharm, Vechta, Germany
xylazine	Rompun®, 2% ad us. vet.	Elanco, Bad Homburg, Germany

NaCl – sodium chloride; WDT – Wirtschaftsgenossenschaft deutscher Tierärzte.

Supplementary Table S5: Buffers used for Western blot.

	Quantitative Ratio	Cat. No.	Supplier
**Lysis buffer**			
IGEPAL CA-630	1%	I3021-50ML	Merck KGaA, Darmstadt, Germany
TRIS-HCl, pH 8.0	50 mM	9090.3	Carl Roth GmbH + Co. KG, Karlsruhe, Germany
NaCl	150 mM	HN00.2	Carl Roth GmbH + Co. KG
NaF	5 mM	S6776-100G	Merck KGaA
Na_3VO_4	1 mM	S6508-10G	Merck KGaA
protease inhibitor cocktail	1 tablet/10 mL lysis buffer	05892791001	Merck KGaA
**1x Running buffer**			
TRIS base	25 mM	4855.5	Carl Roth GmbH + Co. KG
glycine	192 mM	3790.2	Carl Roth GmbH + Co. KG
SDS	0.1%	2326.2	Carl Roth GmbH + Co. KG
**Tris-buffered saline with Tween® 20 (TBS-T) buffer**			
TRIS-HCl	15.2 mM	9090.3	Carl Roth GmbH + Co. KG
TRIS base	4.6 mM	4855.5	Carl Roth GmbH + Co. KG
NaCl, pH 7.6	150 mM	HN00.2	Carl Roth GmbH + Co. KG
Tween® 20	0.01%	P9416	Merck KGaA

Cat. No. – catalogue number; IGEPAL – octylphenoxy poly(ethyleneoxy)ethanol, NaCl – sodium chloride; NaF – sodium fluoride; Na_3VO_4 – sodium orthovanadate; SDS – sodium dodecyl sulfate; TRIS – tris(hydroxymethyl)aminomethane; TRIS-HCl – TRIS hydrochloride; Tween® 20 – polyoxyethylene (20) sorbitan monolaurate.

Supplementary Table S6: Primary and secondary antibodies used for immunoblot analyses.

Antibody	Dilution	Incubation time	Cat. No.	Supplier
rabbit monoclonal anti-HO-1	1:2400 in TBS-T	Overnight, 4°C	70081S	Cell Signaling Technology Inc., Danvers, USA
anti-rabbit IgG, HRP-linked	1:3000 in TBS-T	1 h, room temperature	7074S	Cell Signaling Technology Inc.

Cat. No. – catalogue number; TBS-T – TRIS-buffered saline with Tween® 20;

Supplementary Table S7: Primary and secondary antibodies used for immunohistochemistry.

Antibody	Dilution	Batch	Cat. No.	Supplier
polyclonal goat anti-KIM-1	1:1000 in TRIS buffer + 1% BSA	KCA0318012	AF1817	R&D Systems, Inc., Minneapolis, USA
monoclonal rat anti-CD31	1:100 in TRIS buffer + 1% BSA	17928/29	DIA-310	Biozol Diagnostica Vertrieb GmbH, Eching, Germany (brand Dianova)
biotinylated anti-goat IgG (H+L)	1:500 in TRIS buffer	ZA0425	BA-5000	Vector Laboratories, Inc., Newark, USA
biotinylated anti-rat IgG (H+L)	1:200 in TRIS buffer + 2% NMS	ZE0201	BA-4001	Vector Laboratories, Inc.

Cat. No. – catalogue number; BSA – bovine serum albumin; NMS – normal mouse serum (P30-0200, PAN-Biotech GmbH, Aidenbach, Germany); TRIS – tris(hydroxymethyl)aminomethane.
